# Detection and characterization of COVID-19 findings in chest CT: Feasibility and applicability of an AI-based software tool

**DOI:** 10.1097/MD.0000000000027478

**Published:** 2021-10-15

**Authors:** Andi Gashi, Rahel A. Kubik-Huch, Vasiliki Chatzaraki, Anna Potempa, Franziska Rauch, Sasa Grbic, Benedikt Wiggli, Andrée Friedl, Tilo Niemann

**Affiliations:** a Department of Health Sciences and Technology, Swiss Federal Institute of Technology, ETH Zurich, 101 Rämistrasse, Zurich, Switzerland; b Department of Radiology, Kantonsspital Baden, 1 Im Ergel, Baden, Switzerland; c Siemens Healthcare GmbH, 3 Siemensstrasse, Forchheim, Germany; d Department of Infectious Diseases, Kantonsspital Baden, 1 Im Ergel, Baden, Switzerland.

**Keywords:** artificial intelligence, computed tomography, COVID-19, decision support, pneumonia

## Abstract

The COVID-19 pandemic has challenged institutions’ diagnostic processes worldwide. The aim of this study was to assess the feasibility of an artificial intelligence (AI)-based software tool that automatically evaluates chest computed tomography for findings of suspected COVID-19.

Two groups were retrospectively evaluated for COVID-19-associated ground glass opacities of the lungs (group A: real-time polymerase chain reaction positive COVID patients, n = 108; group B: asymptomatic pre-operative group, n = 88). The performance of an AI-based software assessment tool for detection of COVID-associated abnormalities was compared with human evaluation based on COVID-19 reporting and data system (CO-RADS) scores performed by 3 readers.

All evaluated variables of the AI-based assessment showed significant differences between the 2 groups (*P* < .01). The inter-reader reliability of CO-RADS scoring was 0.87. The CO-RADS scores were substantially higher in group A (mean 4.28) than group B (mean 1.50). The difference between CO-RADS scoring and AI assessment was statistically significant for all variables but showed good correlation with the clinical context of the CO-RADS score. AI allowed to predict COVID positive cases with an accuracy of 0.94.

The evaluated AI-based algorithm detects COVID-19-associated findings with high sensitivity and may support radiologic workflows during the pandemic.

**Key points**

- AI-assessment showed significant differences between rt-PCR-positive patients and asymptomatic patients.
- The inter-reader reliability of CO-RADS scoring was almost perfect.
- AI-assessment and human scoring differed significantly but showed solid clinical correlations.

## 1 Introduction

The on-going COVID-19 pandemic confronts health-care professionals worldwide with unprecedented clinical and operational challenges. These challenges will result in significant changes to health-care systems in general and radiology departments in particular.^[1–8]^ The increased number of artificial intelligence (AI) applications that have become available during the pandemic has driven innovation.^[9]^ Chest computed tomography (CT) imaging currently plays an important role in diagnosing COVID-19.^[1–8]^

The applications of AI in the clinical setting are manifold during this pandemic. Some projects have used AI to enable a contactless imaging workflow that reduces the infection risk for staff and patients.^[9–11]^ Other projects have implemented AI-based tools for automatic segmentation and labelling of regions of interest to reduce radiologists’ workload and to increase the sensitivity and detection rate.^[9,12]^ Other on-going projects use AI-based software tools to improve differential diagnosis and increase specificity.^[9]^ The main focus of current AI based studies for COVID recognition in chest CT is to detect disease in affected lungs, to accurately segment anatomic structure and lung opacities and to classify severity of disease.^[13,14]^ This is basically done using machine learning and deep learning approaches. Convolutional neural networks or different models produced from convolutional neural networks are widely encountered since they contain both feature extraction and classification stages.^[13]^ Recommendations by various societies reflect the focus on implementation of AI in both predictive and prognostic decision support systems. However, the use of AI on CT scan data for screening or first-line diagnostic tests for COVID-19 is still under debate.^[15]^ Nevertheless, the European Society of Medical Imaging Informatics officially supports efforts such as the Imaging COVID-19 AI European Initiative (imagingcovid19ai.eu).^[16]^

Recently, Siemens Healthineers (Forchheim, Germany) has released a prototype of an AI-based software tool for chest CT analysis (*syngo*.via CT Pneumonia Analysis prototype). The algorithm is designed to automatically identify and quantify abnormal tomographic patterns in the lungs from chest CT, to reduce the diagnostic workload per patient, and to improve the detection and management of possible COVID-19-cases. The algorithm was trained using international multicentre datasets.^[17]^

The primary objective of this study was to evaluate the feasibility and applicability of an AI-based software prototype to detect COVID associated lung abnormalities in chest CT. In clinical routine the prototype was applied in patients with known COVID-19 disease and an asymptomatic control cohort. Additional human assessment was evaluated. Performance of the AI prototype and human scoring for assessment of typical COVID abnormalities was evaluated.

## 2 Materials and methods

This study was approved by the local Ethics Committee (EKNZ Nr. 2020–00955), and patients’ written informed consent was waived.

### 2.1 Setting

The retrospective analysis of feasibility was situated at a large hospital with a huge emergency department, classified as major hospital for COVID treatment.

Based on the regulations of the Swiss Federal Council and experts’ recommendations, our institution's pandemic board implemented a general screening method from April 8^th^ to 27^th^ (ie, during the height of the pandemic). In order to increase the detectability of real-time polymerase chain reaction (rt-PCR)-“silent” infected candidates for surgical treatment preoperative chest CT of the patients at a time-point of maximum 24 hours before surgery was recommended by experts.^[18–20]^ This was performed independently of the presence of respiratory symptoms. Dose-reduced chest CT was administered to candidates for emergency surgery for perioperative risk assessment regarding COVID-19-associated findings (group B).

### 2.2 Patient cohorts

**Figure 1:**
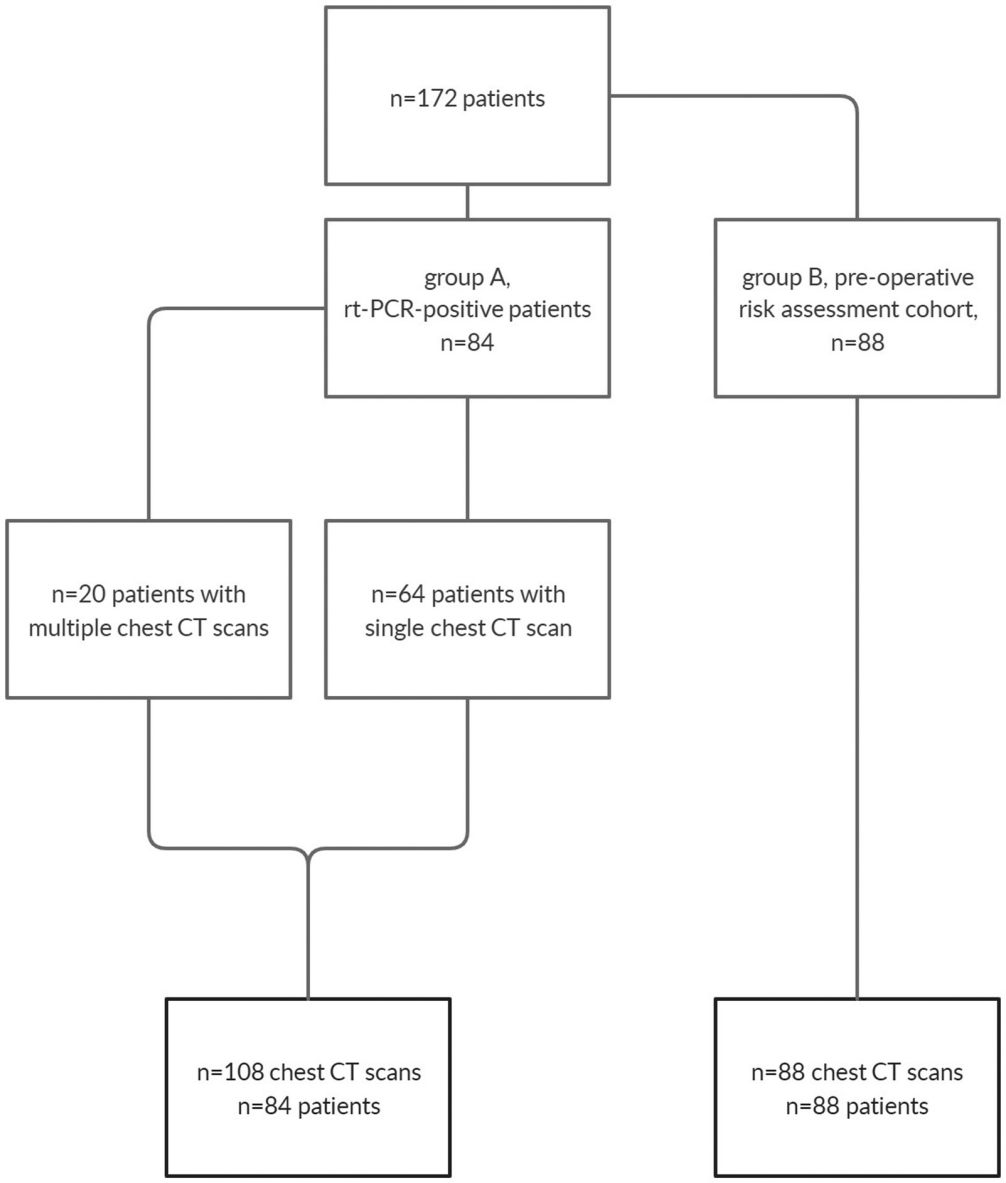
Flowchart showing the retrospective acquisition of patient data from n = 172 patients and their subdivision into groups A and B. CT = computed tomography, rt-PCR = real-time polymerase chain reaction.

**Figure 2:**
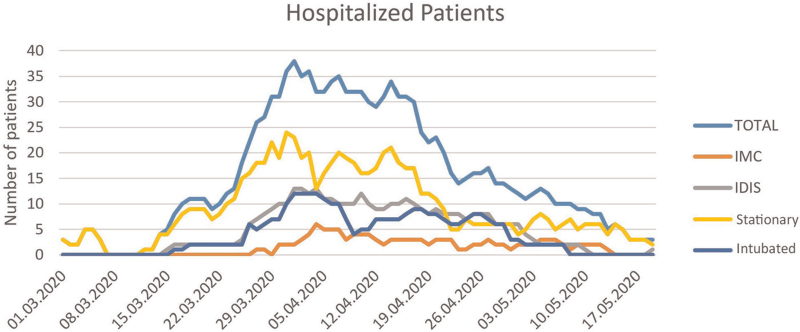
Hospitalised patients at our institution. The X-axis shows the date, ranging from March 1^st^ to May 17^th^. The Y axis shows the number of patients that were hospitalised in our hospital in Switzerland at that specific time point. Blue: total, orange: intermediate care unit, grey: interdisciplinary intensive care unit, yellow: stationary, green: intubated. Our analysis includes CT data acquired between March 18^th^ and April 30^th^. IMC = intermediate care unit, IDIS = interdisciplinary intensive care unit.

The study population consisted of 2 groups consecutively enrolled from March 18^th^ to April 27^th^ (Fig. 1): a symptomatic cohort that underwent chest CT for evidence of COVID-19 findings (group A) and a cohort consisting of asymptomatic patients that underwent chest CT for perioperative risk assessment (group B). All patients in group A were tested positive with SARS-CoV-2-specific rt-PCR testing prior to CT scanning. Figure 2 shows that the chosen timeframe for screening coincides with the peak of the pandemic at our institution so far. The patients in group B also participated in another publication about perioperative risk assessment during the COVID pandemic that has been submitted for publication. The patients of group B were not systematically tested with rt-PCR since they were asymptomatic or would not have timely results of rt-PCR testing prior to CT scanning. All patients of group B had been scheduled for emergency surgery, not allowing waiting for rt-PCR test results.

Our database was retrospectively searched for all consecutive chest CT examinations for both groups. The inclusion criteria were age ≥18 years at the time of the CT scan, proven COVID disease for group A and referral for dedicated perioperative risk assessment for group B.

Several patients from group A underwent more than 1 chest CT scan, which were all included in the study.

### 2.3 Image acquisition

Chest CT of group A was performed using a dedicated Siemens Definition AS + scanner (Siemens Healthineers, Forchheim D). This scanner was used solely for COVID-19 assessment to minimise the risk of cross-infection and contamination. The acquisition parameters were 120 ref.Kv, 60 ref.mAs, CarekV and CareDose activated, rotation time 0.3 second, and pitch 1.45.

Group B was scanned using a Siemens Definition Flash scanner. The acquisition parameters were 120 ref.Kv, 65 ref.mAs, CarekV, and CareDose activated, rotation time 0.28 second, and pitch 1.5.

CTDI_Vol_ was documented for both groups as an indicator of the radiation dose.

### 2.4 Image analysis

Image analysis was performed in Picture Archiving and Communications Systems (GE Centricity version I6, GE Healthcare, Chalfont, St Giles, UK). Two readers who were blinded regarding clinical information and rt-PCR results (TN, with 15 years of experience in chest CT, and AP, with 3 years of experience) independently evaluated both groups’ images. The chest CT images were rated according to the COVID-19 reporting and data system (CO-RADS) scheme.^[21]^ Cohen kappa coefficient and weighted kappa coefficient^[22]^ were calculated to assess inter-reader reliability. Discrepancies between the 2 readers were resolved by a third reader (VC, with 3 years of experience). The readers were able to adjust the image size and windowing during image review to facilitate evaluation of soft tissues and pulmonary parenchyma.

### 2.5 Image analysis using AI-based software tool

**Figure 3:**
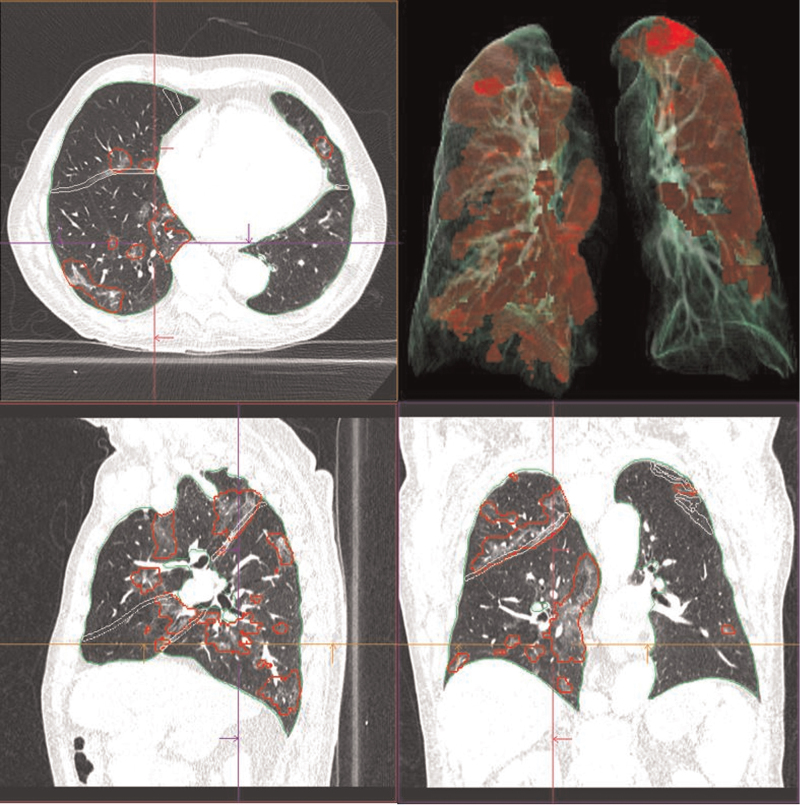
Coronal, axial, and sagittal slices of lungs and 3D reconstruction as an example of visual output of AI-based software tool (*syngo*.via CT Pneumonia Analysis prototype). AI software analysis of a 78-year-old male patient presenting to the emergency ward with fever and increased tiredness. This patient was rt-PCR-positive for SARS-CoV-2.

**Figure 4:**
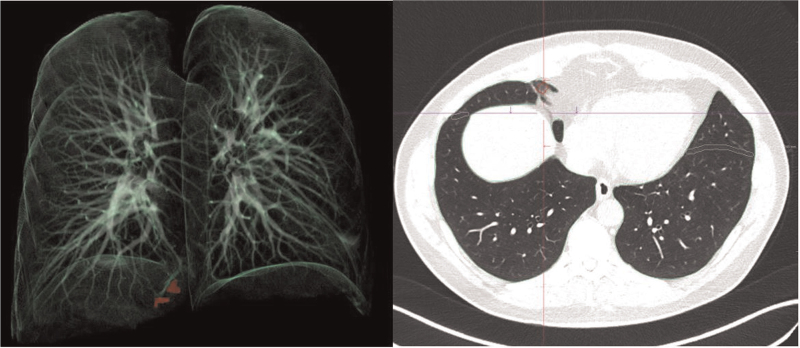
3D reconstruction and axial slices of the lungs (*syngo*.via CT Pneumonia Analysis prototype). Analysis of a 58-year-old male patient who had undergone chest CT as preoperative measurement (for hip replacement) to reduce infection risk in the pandemic setting. This patient was rt-PCR-negative for SARS-CoV-2.

AI-based software analysis was performed with the *syngo*.via CT Pneumonia Analysis prototype (Version 1.0.4.2, Siemens Healthineers, Forchheim, Germany). Figures 3 and 4 show examples of the *syngo*.via prototype's output. The algorithm automatically delineates the airspace opacities using a convolutional neural network trained with data that had been manually labelled by clinical experts. Technical details for the evolution of the algorithm have been described before.^[17,23]^ It provides opacity scores, percentages of opacity (relative to overall lung volume), and percentages of high opacity (relative to overall lung volume). To distinguish between ground glass opacities and consolidations, a threshold of −200 HU is applied inside the detected airspace opacities. Areas denser than −200 HU are considered as high opacities. The calculation of the opacity score is based on the paper of Bernheim et al.^[24]^ The algorithm is based on advanced deep machine learning methods, and its overall performance metrics are area under the curve (AUC) of 0.9 and sensitivity and specificity of 86% and 81%, respectively.^[17]^

Each scan was evaluated for opacity score (range 0–20), percentage of opacity (range 0%–100%), and percentage of high opacity (range 0%–100%). Additionally, the AI-based software tool decided whether cases were “affected” (yes or no) based on its findings. Metrics for the parameter evaluated have been described before in detail.^[17]^

### 2.6 Extraction of clinical data

Our institution's internal database (KISIM Clinical Information System, Cistec, Zurich, Switzerland) was retrospectively accessed for the extraction of clinical data such as SARS-CoV-2-specific rt-PCR results, age, sex, and body height and weight for BMI calculation.

### 2.7 Statistical analysis

Results were expressed as means ± standard deviations for continuous variables and frequencies and percentages for categorical variables. R (R Foundation for Statistical Computing, Vienna, Austria, URL: http://www.R-project.org/)^[25]^ was used for all statistical analyses. Inter-reader reliability was assessed by calculation of Cohen kappa coefficients. The Wilcoxon–Mann–Whitney test was performed for comparison of the opacity score, percentage of opacity, and percentage of high opacity between the 2 groups. For comparisons between patients with different CO-RADS scores (independently from the groups), analyses of the 3 variables extracted from the *syngo.*via Kruskal–Wallis tests were performed to explore the differences in terms of opacity score, percentage of opacity and percentage of high opacity among the distinct CO-RADS score categories. Spearman correlation analysis were performed to evaluate the correlations between the human observations using CO-RADS scoring and the features extracted from AI assessment by the software. Posthoc analysis using the Dunn test was performed to detect significant differences between the distinct groups.

### 2.8 Predictive modelling of COVID infection

At the beginning of the first wave of the ongoing pandemic we struggled on how to define a positive or negative COVID patient. Since the positive/false negative rt-PCR results were a major problem then, it was under discussion if positive should be defined as rt-PCR positive only, as a typical clinical constellation despite negative rt-PCR or in combination with highly suspicious CT images (CO-RADS 5). Therefore we applied the Random Forrest machine learning model to predict the COVID infection status from the deep learning-based image features. We used the function randomForest from the R package randomForest v4.6–14 with the default parameters. To exclude false positive rt-PCR results we used only samples that had a CO-RADS score of 5 (high suspicion) for COVID positivity in group A. Accordingly to exclude false negative cases in group B, that is, asymptomatic COVID patients, we only used samples that had a CO-RADS score of 1 (normal). After such filtering there were 126 patients with a high quality ground truth (63 labelled COVID positive and 63 labelled COVID negative).

Random forest was applied to predict if the patients are COVID positive. The variables OPASCR, PEROPAC, and PEROHOPAC were used for this analysis and a fivefold cross validation was performed for evaluation of the prediction. Receiver operator characteristic curves (ROC), the area under the ROC, sensitivity/specificity and positive/negative predictive values were calculated to test the performance of the classification. A 95% confidence interval (CI) for the area under the ROC was calculated by nonparametric bootstrapping (R = 999). The accuracy was computed along with a 95% CI.

## 3 Results

### 3.1 Patient cohorts

Overall, n = 172 patients were included in the study, divided into 2 cohorts (COVID-19 group A: n = 84, with a total of n = 108 chest CT scans; asymptomatic group B: n = 88). Twenty patients in group A underwent more than 1 CT scan, resulting in the total numbers of 108 and 88 CT scans in groups A and B, respectively. An overall of 8 patients of group B was tested with rt-PCR in the perioperative workup. Subsequent repeated rt-PCR was performed in 7 of these and was negative in all of them (Tables 1 and 2).

**Table 1:** Descriptive data of the study population (n = 172).

	Group A	Group B
Number of patients – n	84	88
Number of chest CT sessions – n	108	88
Age – Mean ± SD (yr)	64.7 ± 16.1	61.3 ± 19.4
Gender		
Male – n (%)	54 (64%)	62 (69%)
Female – n (%)	30 (36%)	29 (32%)
BMI – Mean ± SD (kg/m^2^)	27.9 ± 6.8	26.1 ± 6.4
SARS-CoV-2 rt-PCR		
No – n (%)	0 (0%)	83 (91%)
Yes – n (%)	84 (100%)	8 (9%)
Positive – n (%)	84 (100%)	0
Negative – n (%)	0 (0%)	8 (100%)

**Table 2:** Descriptive data from chest CT evaluations of group A (rt-PCR-positive for COVID-19, n = 108) and group B (preoperative cohort, n = 88) according to the CO-RADS grading system^[17]^.

Group A CO-RADS grade	Suspicion	n (%)	Opacity score (mean ± SD)	Percentage of opacity (mean ± SD)	Percentage of high opacity (mean ± SD)
1	Very low	2 (2%)	3.00 ± 2.83	0.48 ± 0.66	0.12 ± 0.17
2	Low	6 (6%)	4.33 ± 1.51	3.93 ± 4.24	0.98 ± 0.98
3	Equivocal	15 (14%)	7.33 ± 3.31	18.8 ± 17.71	4.79 ± 4.90
4	High	22 (20%)	8.09 ± 4.58	24.39 ± 24.27	7.17 ± 9.57
5	Very high	63 (58%)	8.33 ± 3.91	26.65 ± 21.84	6.01 ± 7.44
Overall	–	108 (100%)	7.82 ± 3.97	23.35 ± 21.68	5.69 ± 7.43

Group B CO-RADS grade	Suspicion	n (%)	Opacity score (mean ± SD)	Percentage of opacity (mean ± SD)	Percentage of high opacity (mean ± SD)
1	Very low	63 (72%)	2.16 ± 2.13	1.32 ± 5.64	0.41 ± 1.89
2	Low	11 (13%)	3.00 ± 1.10	0.76 ± 0.68	0.10 ± 0.09
3	Equivocal	10 (11%)	4.00 ± 3.02	5.07 ± 12.30	0.99 ± 2.51
4	High	3 (3%)	4.33 ± 2.31	7.81 ± 10.73	1.17 ± 1.81
5	Very high	1 (1%)	19	84.18	28.95
Overall	–	88 (100%)	2.74 ± 2.86	2.82 ± 10.98	0.79 ± 3.55

### 3.2 Image analysis

**Figure 5:**
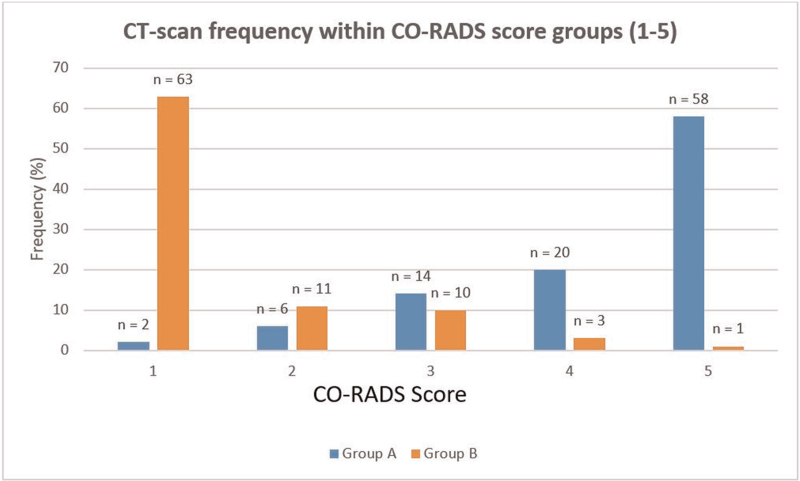
Numbers of CT-scans in groups A and B, subdivided into CO-RADS score groups (rated by human evaluation). CO-RADS = COVID-19 reporting and data system, CT = computed tomography.

Figure 5 depicts the classification of scans from both groups into CO-RADS 1 to 5. There was absolute agreement amongst the readers about 155 (79.08%) of the 196 observations. Cohen kappa for both groups together was 0.72 (95% CI: 0.65–0.80). Cohen kappa group A were 0.62 (95% CI: 0.50–0.75), and the results for group B were 0.65 (95% CI: 0.51–0.80), respectively

### 3.3 Image analysis using AI-based software tool

**Figure 6:**
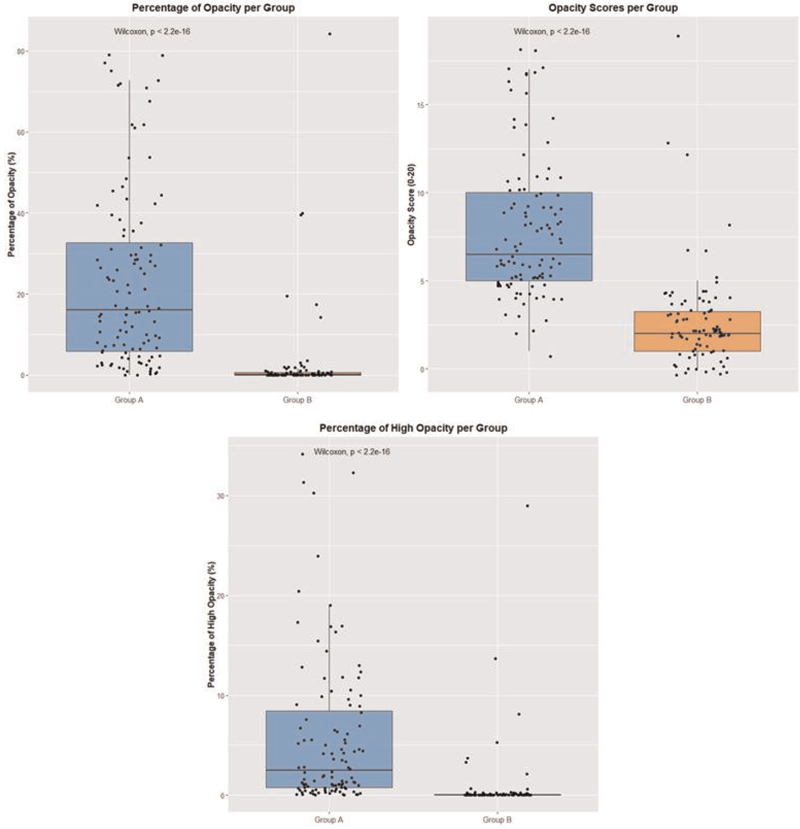
Boxplots of opacity score (left top), percentage of opacity (right top), and percentage of high opacity (bottom) between groups A and B. All 3 variables differed significantly between groups A and B (Wilcoxon–Whitney–Mann test: *P*-values < .01 for all three comparisons).

The opacity scores, percentages of opacity, and percentages of high opacity were 7.82 ± 3.97, 23.35 ± 21.68, and 5.69 ± 7.43 for group A and 2.74 ± 2.83, 2.82 ± 10.98, and 0.79 ± 3.55 for group B, respectively. All 3 variables differed significantly between groups A and B (Wilcoxon–Mann–Whitney *P* < .01 for all 3 variables, Fig. 6).

Figure 6 shows that most scans from group B had very low percentages of high opacity, with 6 outliers having higher percentages (>10%) of affected lung tissue.

### 3.4 Comparison between CO-RADS and AI analysis

**Figure 7:**
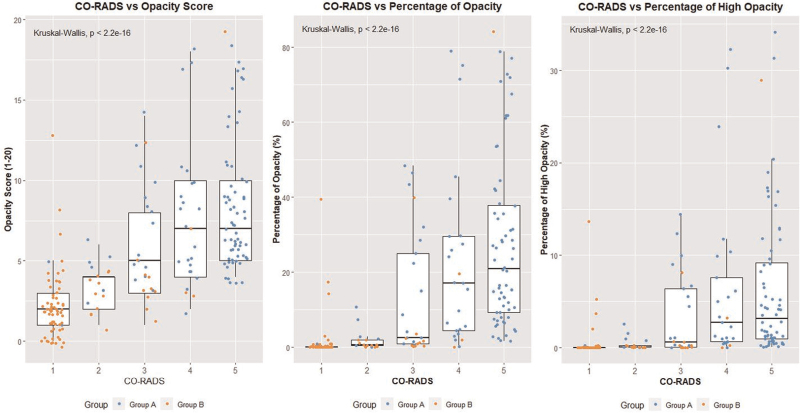
Boxplots of CO-RADS vs opacity score (left), percentage of opacity (middle), and percentage of high opacity (right). Kruskal–Wallis test: *P*-values < .01 for all 3 comparisons. CO-RADS = COVID-19 reporting and data system.

There were significant differences in terms of opacity score, percentage of opacity, and percentage of high opacity among the CT scans with different CO-RADS scores (Kruskal–Wallis tests for all 3 variables: *P* < .01). Posthoc analysis showed that all 3 variables differed significantly between all CO-RADS score pairs (Dunn tests for all CO-RADS pairs for all 3 *syngo*.via variables: *P* < .05) (Fig. 7). Spearman correlation for analysis of correlation between human scoring and features extracted by the software showed strong correlation for all patients for opacity score (0.74), for percentage of opacity (0.78) and for percentage of high opacity (0.73). Separate analysis for both groups showed low correlation for opacity score (0.24), percentage of opacity (0.31), and percentage of high opacity (0.18) for group A and moderate correlation for opacity score (0.43), percentage of opacity (0.53), and percentage of high opacity (0.51) for group B.

Figure 7 shows that CT scans rated with lower CO-RADS scores (ie, 1 or 2) had lower percentages of high opacities. In group B, 63 (72%) of the CT scans were classified as CO-RADS 1, bearing very low suspicion for COVID-19 because there were only few or no ground glass opacities.

### 3.5 Predictive modelling of COVID infection

**Figure 8:**
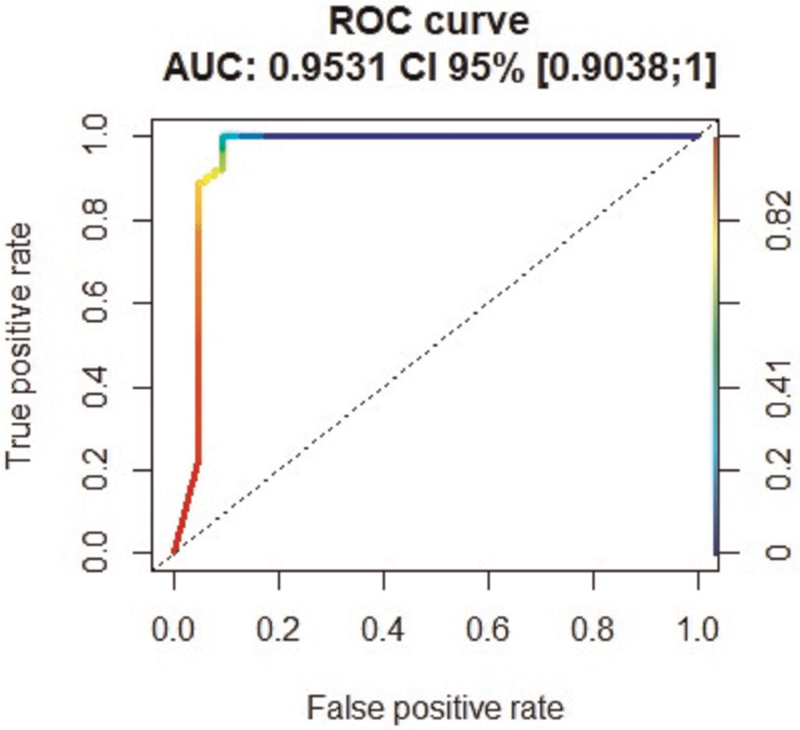
ROC analysis of the random forest prediction. The colour indicates the cutoff that turns the scoring output of the random forest classifier into a binary class decision. AUC = area under the curve, ROC = receiver operator characteristic curve.

We predicted if a patient was COVID positive by using the 3 variables from the AI analysis (opacity score, percentage of opacity, and percentage of high opacity). Random forest, an ensemble learning method, was used for classification of the patients. Table 3 shows the confusion matrix of the predicted labels from the random forest analysis compared with the ground truth (see Section 2). Sensitivity/specificity was 0.97/0.90, respectively. Positive/negative predictive value was 0.91/0.97, respectively. The accuracy was 0.94 (95% CI: 0.88–0.97). A ROC analysis of the random forest prediction was preformed and the AUC was calculated. AUC: 0.95 (95% CI: 0.90–1) (Fig. 8).

**Table 3:** Confusion matrix of the predicted labels from the random forest analysis compared with the ground truth.

	COVID−	COVID +
Prediction −	57	2
Prediction +	6	61

## 4 Discussion

The controversy surrounding the diagnostic value of rt-PCR and chest CT for COVID-19 cases is well-known.^[1,3,5,8]^ Although there have been attempts to improve the diagnostic procedure using rt-PCR,^[26]^ chest CT has claimed a special position in the early diagnostic procedure of early COVID-19 cases because of its ability to detect intrapulmonary changes during early disease stages, when rt-PCR tests might show false-negative results.^[3]^ The sensitivity of chest CT has been reported to be around 86% to 96%.^[2]^ According to the ESR/ESTI advice paper and recommendations of the French Society of Thoracic Imaging, unenhanced chest CT is currently indicated for patients presenting with dyspnea, polypnea or desaturation in order to refer them to “COVID” or “non COVID” wards, pending RT-PCR results.^[27]^

Thus, analysis of chest CT images can support clinical suspicion of COVID-19 positivity and be an indication for repeated rt-PCR-tests,^[28,29]^ as the false-negative rate of rt-PCR tests is highly variable throughout the course of the disease.^[30]^ Still, the difficult distinction between COVID-19 and other viral pneumonia findings in the lung has been reported as the main reason for the low specificity of chest CT, with results ranging 25% to 33%.^[2,5]^ However, a recent low-dose CT study achieved 93% to 94% specificity,^[7]^ depending on disease stage. Our results are in line with other published results for the diagnostic performance of human reading that was reported as an AUC of 0.91 by Prokop et al^[21]^ using the CO-RADS score. Other authors reported an analogous distribution of CO-RADS scoring and reader agreement compared with our results.^[31]^

A similar performance for the AI algorithm compared to our results was reported in the validation study by Georgescu et al,^[17]^ the authors reported a best performance with an AUC of 0.9 in their population. Recent meta-analysis describe an AUC of 99.87% and 0.96 to 0.99, respectively for all mathematic models.^[13,14]^ Approaches that aim to detect COVID disease showed a pooled AUC of 0.949^[13]^ that is in line with the performance of our algorithm.

The prototype algorithm evaluated was designed to automatically identify and quantify abnormal tomographic patterns in the lungs in the context of COVID-19.^[17]^ It is based on clinically interpretable severity metrics computed from automated segmentation of abnormal regions in chest CT images, as well as a black-box approach using an advanced deep learning system.^[17]^ Even if the paper presented by Georgescu et al^[17]^ represents a rather technical manuscript that focuses on the implementation and validation of the AI algorithm, there is a certain overlap in the study population characteristics and characteristics of the control group chosen in our methodology. While the authors tested the algorithm in a COVID+ group (n = 100), only partly confirmed by rt-PCR. Their COVID− group consisted of healthy patients (n = 34) and patients with known interstitial or other pneumonias (n = 60). Our COVID+ group consisted of 108 rt-PCR+ symptomatic patients. Our COVID− control group was a special collective that underwent emergency surgery during the height of the pandemic and was scheduled for perioperative risk assessment (n = 88). All patients were clinical asymptomatic for COVID disease. The group around Georgescu et al^[17]^ used a variety of different scanners, acquisition and reconstruction parameters. In our population, all patients of each group were scanned on the same scanner with standardized parameters.

Our results suggest that COVID disease can be predicted based on AI-derived CT image features. The routine application of AI-based software tools can be considered in pandemic situations to quickly confirm or rule out pulmonary affection in patients without or with subtle chest CT findings. No patients in group A and 14 out of 88 patients (16%) in group B had an Opacity score of <1, suggesting that this AI-based software tool has a high detection rate of ground glass opacity findings on chest CT. This correlates with the weaker correlation for group A compared with group B for CO-RADS and all AI-parameters that might reflect a tendency of the software towards over-sensitivity. The plots in Figure 7 suggest that even if COVID-19 cannot be fully excluded, the AI-based software tool is good enough to detect pulmonary involvement in disease as could be shown by an AUC of 0.95 and an accuracy of 0.94. Thus, it is a candidate as an early and rapid screening method to rule out the need for enhanced protective measures against cross-infection in times of resource scarcity, such as at the beginning of the present pandemic. As stated in the validation study the algorithm evaluated does not generate results with 100% accuracy as can be seen in Fig. 3 that nicely demonstrates areas of ground glass opacities omitted by the segmentation software. Hence the possibility of false negative estimation must be taken into account when controlling the AI generated results, especially in region at risk for errors such as paravertebral dystelectasis.

Our study has several limitations. First, 2 different scanners were used for the two cohorts to mitigate the infection risk for the patients. Thus, the acquired image quality used for subsequent AI-based postprocessing was not absolutely identical, but patient centered dose modulation algorithms were activated for both scanners. Second, it included a population assessed during the acute phase of the SARS-CoV-2 outbreak, which has a public prevalence that is still unidentified until today. Because the diagnostic tests for SARS-CoV-2 do not have 100% specificity and sensitivity, SARS-CoV-2-positive patients might have been included in the COVID-19-negative group (group B). Reasonable estimates at least for an upper limit of the local incidence rate could help to derive the probability of observing false negative events. Third, our study presents a further variance of AI based segmentation of COVID disease in chest CT but does not support the recently proposed AI pathway of combined clinical background and CT findings. However our data may contribute for further optimization of AI based pattern recognition to enhance the evolution of current AI strategies.

Forth to date the AI algorithm aforementioned was trained using imbalanced datasets resulting in a confusion matrix that is highly efficient in distinguishing positive CT from normal lung CT but that is poor in distinguishing between COVID associated abnormalities and other causes for opacities. Application of the current algorithm out of heights of the pandemic should be handled with care due to an overlap of imaging features of differential diagnosis.

Fifth our study design was of explorative character and no confirmative approach.

AI-powered analysis of CT images has the potential to reduce the growing burden on radiologists during the pandemic, speed up their reading time, and support accuracy. The algorithm may provide support of patient triage, diagnosis (in combination with rt-PCR tests and epidemiological risk), assessment of severity and progression, and response to therapeutic alternatives in patients exhibiting COVID-19 symptoms. While current studies clearly demonstrate the high performance of AI based pattern recognition as scoring for COVID disease,^[32]^ recent research propagates combined AI models that integrate both CT imaging and clinical information to enhance the accurate diagnosis of COVID disease.^[33]^

## 5 Conclusion

In conclusion, to our knowledge, this is the first effort to deploy the algorithm in the routine clinical practice of a Radiology Department during the ongoing COVID-19 surge. The current results confirm that this highly effective AI algorithm for rapid identification of patients with COVID-19 could be helpful in further waves of the current pandemic. The proposed AI model could be a useful screening tool for quickly ruling out infectious diseases such as COVID-19 that does not require radiologist input and supports rapid patient triage during local peak pandemic stages.

## Acknowledgments

We thank Richard Lipkin, PhD, from Edanz Group (https://en-author-services.edanzgroup.com/) for editing a draft of this manuscript.

We thank Lars Bosshard and Michael Prummer of NEXUS Personalized Health Technologies, ETH Zürich, and Swiss Institute for Bioinformatics, Zürich, for support with predictive modeling and statistics.

## Author contributions

**Conceptualization:** Andi Gashi, Rahel A. Kubik-Huch, Vasiliki Chatzaraki, Benedikt Wiggli, Tilo Niemann.

**Data curation:** Vasiliki Chatzaraki, Tilo Niemann.

**Formal analysis:** Andi Gashi, Vasiliki Chatzaraki, Anna Potempa, Tilo Niemann.

**Funding acquisition:** Rahel A. Kubik-Huch.

**Investigation:** Andi Gashi, Vasiliki Chatzaraki, Benedikt Wiggli, Andrée Friedl, Tilo Niemann.

**Methodology:** Andi Gashi, Vasiliki Chatzaraki, Benedikt Wiggli, Tilo Niemann.

**Project administration:** Rahel A. Kubik-Huch, Vasiliki Chatzaraki, Andrée Friedl, Tilo Niemann.

**Resources:** Rahel A. Kubik-Huch, Sasa Grbic.

**Software:** Franziska Rauch, Sasa Grbic.

**Supervision:** Vasiliki Chatzaraki, Tilo Niemann.

**Validation:** Vasiliki Chatzaraki.

**Visualization:** Franziska Rauch, Tilo Niemann.

**Writing – original draft:** Vasiliki Chatzaraki, Anna Potempa, Tilo Niemann.

**Writing – review & editing:** Rahel A. Kubik-Huch, Benedikt Wiggli, Andrée Friedl, Tilo Niemann.
